# Morphological changes in polyester prosthesis geometry after open aortic repair

**DOI:** 10.1186/s12872-025-04851-0

**Published:** 2025-06-02

**Authors:** Joseph Kletzer, Wanda Buechner, Martin Czerny, Roman Gottardi, Tim Berger, Aleksandar Dimov, Mario Lescan, Maximilian Kreibich, Stoyan Kondov

**Affiliations:** 1 Department of Cardiovascular Surgery, University Hospital Freiburg Heart Centre, Freiburg, Germany; 2 Faculty of Medicine, University of Freiburg, Freiburg, Germany; 3 University Hospital Freiburg– Heart Centre, Südring 15, 79189 Bad Krozingen, Germany

**Keywords:** Aortic surgery, Vascular prosthesis, Computed tomography angiography, Dacron graft, Geometry changes

## Abstract

**Objectives:**

Aim of this study was to assess geometrical changes of implanted Dacron grafts following open surgical ascending aortic replacement.

**Methods:**

Geometrical Dacron graft changes were analysed during the postoperative follow-up of 215 who received ascending aortic surgery between 02/2010 and 12/2020. Data was analysed using a linear mixed effects model over long-term follow-up.

**Results:**

One-hundred forty-five (67%) patients of our cohort were male, with a median age of 61 years 52–71.5). Most patients had a history of hypertension (79%). Proximal diameter of the implanted grafts grew at a rate of 0.251 cm (0.181–0.328 cm; *p* < 0.001) per year. In contrast, distal diameter stayed constant over time (0.019 cm; -0.048–0.135 cm; *p* = 0.366), while prosthesis length measured at the centreline decreased at a rate of -0.835 cm (-0.969 cm - -0.581 cm; *p* < 0.001) per year.

**Conclusion:**

In contrast to the native aorta, Dacron grafts seem to decrease in length over time. At the same time, there is a significant increase in proximal diameter. The specific dimensional changes, and differences to the nominal graft dimensions, should be considered at the time of graft implantation to ensure a durable platform for secondary aortic interventions.

**Clinical trial number:**

Not applicable.

**IRB:**

04/02/2021 (No. 20-1302)

**Supplementary Information:**

The online version contains supplementary material available at 10.1186/s12872-025-04851-0.

## Introduction

Since its first description by Volodos et al. in 1986 [1], endovascular aortic repair has been gaining ground on open surgery in the treatment of thoracic descending aortic repair. However, open surgery using primarily woven or knitted Polyethylene terephthalate, otherwise known as dacron, grafts is still the first line treatment for most aortic disease, according to current guidelines [2]. Open technique shows lower rates of long-term complications leading to aortic re-interventions and re-do surgery, further cementing its central place in the treatment of most aortic disease.

While the inception of endovascular treatment has sparked a wave of research looking into the behaviour of the native aorta, as understanding changes in aortic geometry is essential for anticipating and preventing complications in endovascular treatment, changes in aortic Dacron grafts were mostly left out. Currently, there is very little data describing how implanted grafts react to the constantly changing hemodynamic of the human body [3]. An important yet often overlooked consideration in aortic surgery is the discrepancy between the nominal size of Dacron grafts, as indicated by manufacturers, and their actual dimensions once implanted and exposed to physiological pressures. This difference can influence outcomes, particularly in hybrid procedures and valve-sparing techniques where precise sizing is paramount. To further the understanding of how our body’s physiology might affect aortic prosthesis and to aid in in planning of future aortic repair, this work aims to analyse changes in Dacron graft diameter and length changes during follow-up and replacement of the ascending, descending and the infrarenal aorta. Our hypothesis is, that over time, not only the geometry of the native aorta, but also that of implanted Dacron grafts changes, raising the necessity for rigorous planning in future endovascular procedures.

## Patients and methods

### Ethics statement

The institutional review board of the University of Freiburg granted IRB approval on the 04/02.2021 (No. 20-1302). Due to the retrospective nature of our study, the need for individual consent was waived.

### Patients

Dacron graft diameter and length changes were analysed during the postoperative follow-up of 215 patients retrospectively. The surgery was performed between 02/2010 and 12/2020 at the University Hospital Freiburg– Heart Centre. Data was analysed using a linear mixed effects model over long-term follow-up. Included were patients treated for degenerative aortic aneurysm, acute and chronic aortic dissection of the ascending thoracic aorta. We excluded patients under the age of 18 years, with scheduled surgery, endovascular procedures, patients with > 1 missing follow-up visit, denying us the possibility of analysing a time series and CTA slice thickness more than 3 mm.

The decision to exclude patients with missing follow-up was made to ensure the integrity of our findings, as follow-up data serve as the outcome variable we aim to predict. Imputation of missing follow-up data was considered but ultimately deemed inappropriate, as it could introduce bias and artificial trends into the analysis. Furthermore, the mechanism of missingness in our study is likely a combination of MAR (missing at random) and MNAR (missing not at random). For example, some patients may have missed follow-up visits due to logistical reasons (MAR), while others may have been unable to attend due to health-related factors (MNAR). Given the challenges of accurately modelling these mechanisms and the risk of unverifiable assumptions, we opted for a conservative approach by excluding patients with insufficient follow-up data. This ensures that our results are based solely on observed data and minimizes the risk of selection bias.

### Data collection and outcome definitions

Data was collected from our electronic patient records. Aortic measurements during follow-up were taken from postoperative CTA using multiplanar reconstruction, assuring correct alignment and always in plane perpendicular to the manually corrected aortic centreline using Deep Unity (Agfa HealthCare N.V., Morstel, Belgium). Measurements were taken during end-diastole. This phase was chosen as it provides a stable and reproducible point in the cardiac cycle for imaging and measurement. Graft length was measured using EndoSize software (Therenva SAS, Rennes, France), which provides precise and standardized tools for dimensional analysis. To minimize variability, all measurements were performed by a single observer, thereby eliminating interobserver variability. Diameters of the Dacron graft were taken at the proximal and distal anastomoses. Length was measured by fitting a centreline. Data was then transferred to our internal aortic database.

Follow-up visits were measured in years, with all patients having the same number of follow-ups recorded. However, the timing of these visits varied between individuals due to differences in adherence to regular follow-up schedules. For some patients, later follow-up visits occurred many years after surgery, while others adhered more closely to standard timelines. To account for this variability, we employed a linear mixed-effects model, which accommodates differences in follow-up timing and allows for robust analysis of longitudinal data.

### Statistical analysis

Data was expressed using mean (SD), number (percentage), or median (interquartile range (IQR)).

The aim of our work was not to compare groups; therefore, no such division was made in the baseline characteristics. RStudio (RStudio: Integrated Development for R. RStudio, Inc., Boston) using R Version (R Core Team (2023). R: A Language and Environment for Statistical Computing. R Foundation for Statistical Computing, Vienna, Austria) was used to perform necessary analyses. The Shapiro-Wilk test and Q-Q plots were used to assess normal distribution of data. Normally distributed data was show using the mean (standard deviation). Non-normal data was presented as median (IQR). Nominal data was displayed as a number (percentage). Visualization of data was done the R-package ggplot2 [4]. To perform linear mixed effects modelling, the lme4 package for R was used [5].

To evaluate diameter and length changes over time we decided to use linear mixed effects modelling. As a fixed effect we investigated time. As random intercepts we chose patient id, initial diameter, and aortic segment of implantation. We also included random slopes by patient id in our model. Singularity of our model was evaluated using the isSingular() function of the lme4 package [5]. Model selection was done based on the Akaike information criterion (AIC). Multiple models were created using additional covariates such as age, sex, height and history of hypertension. The models were then compared using the AIC and the best model was chosen. The assumption of linearity was checked graphically using a scatter plot and smoothed interpolation of the general trend of the data. Homogeneity of variance was tested visually using a fitted vs. residual plot. Normal distribution of residuals was evaluated using a QQ-Plot, as well as the Shapiro-Wilks test. In the case of modelling distal diameter and length, Shapiro-Wilks test yielded p-values below *p* = 0.05, rejecting the assumption of normal distribution of residuals. However, as QQ-Plots revealed minimal skewedness in the lower-most and upper-most residuals, we decided against applying any transformation to our data due to the general robustness of linear mixed effects models to such violation [6]. Additionally, we wanted to prioritize interpretability, which would have been impeded when relying on transformation. Results of estimates are presented as cm per year together with a 95% confidence interval (CI). A p-value of < 0.05 was deemed statistically significant. Robustness of our models was checked using sensitivity analysis omitting outlying residuals of more than 2.5 standard deviations, as well as high leverage datapoints with more than two times the mean leverage. Results can be found in the Supplemental Materials (Table S1).

## Results

### Baseline characteristics

In our cohort of 215 patients, 145 (67%) were male. The median age was 61 years (52–71.5), with a median body mass index (BMI) of 25.95 (23.98–29.15). The majority, namely 169 (79%), of our patients had a history of hypertension. In total, 85 (40%) had a history of smoking, and 60 (28%) were diagnosed with dyslipidaemia. A total of 17 (7.9%) of our patients had bicuspid aortic valves. The overwhelming majority (98%) of our patients were operated on for dilatative aortic disease. A detailed account of baseline characteristics can be found in Table 1.

**Table 1:** Baseline characteristics of the studies cohort; BMI: body mass index; COPD: chronic obstructive pulmonary disease; IQR: interquartile range

Characteristic	*N* = 215^*1*^
Age (years)	61.00 (52.00, 71.50)
Sex (male)	145 (67%)
Height (cm)	175.00 (169.25, 180.75)
Weigth (kg)	80.00 (72.00, 93.75)
BMI	25.95 (23.98, 29.15)
History of Hypertension	169 (79%)
Diabetes Mellitus	8 (3.7%)
History of smoking	85 (40%)
History of alcohol abuse	2 (0.9%)
Dyslipidemia	60 (28%)
Aortic aneurysm present	210 (98%)
Aortic dissection present	93 (43%)
Bicuspid aortic valve	17 (7.9%)

^*1*^Median (IQR); n (%)

### Operative details

In total, 51 (24%) of our patients received a valve bearing conduit. A median of 28 mm (26–29.5) aortic prostheses were used. Median initial prosthesis length was 57.10 (45.95–70.25). Valve sparing aortic root replacement was performed in 20 patients (9.3%). All of these were done using the David technique. In 36 patients (17%) more than one Dacron graft was used during surgery. The aortic arch was at least partially replaced in 47 (22%) patients. The most commonly used prosthesis size was 28 mm (34%). Table 2 describes these surgical characteristics in detail.

**Table 2:** Intraoperative characteristics of the study cohort; IQR: interquartile range

Characteristic	Ascending, *N* = 215^*1*^
Conduit	51 (24%)
Graft specification	
AlboGraft (Le Maitre)	16 (8.7%)
Gelweave (Terumo)	167 (91%)
Uni-Graft (Braun)	1 (0.5%)
Initial proximal diameter (mm)	28.00 (26.00, 29.50)
Initial distal diameter (mm)	28.00 (26.00, 28.00)
Initial prosthesis length (mm)	57.10 (45.95, 70.25)
More than one dacron graft used	36 (17%)
Valve sparring aortic root reconstruction	20 (9.3%)
Aortic arch procedure	47 (22%)
Frequency of graft sizes	
21 mm	3 (1.4%)
22 mm	3 (1.4%)
23 mm	12 (5.6%)
24 mm	6 (2.8%)
25 mm	13 (6.0%)
26 mm	35 (16%)
27 mm	12 (5.6%)
28 mm	73 (34%)
29 mm	4 (1.9%)
30 mm	38 (18%)
31 mm	3 (1.4%)
32 mm	9 (4.2%)
34 mm	4 (1.9%)

^*1*^n (%); Median (IQR)

### Postoperative follow-up

**Fig. 1:**
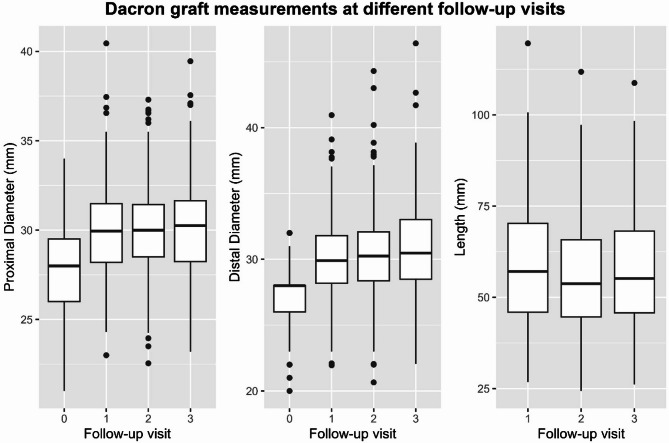
Boxplot visualizing measurements in mm against number of follow-up visit. Black dots delineate outliers

**Fig. 2:**
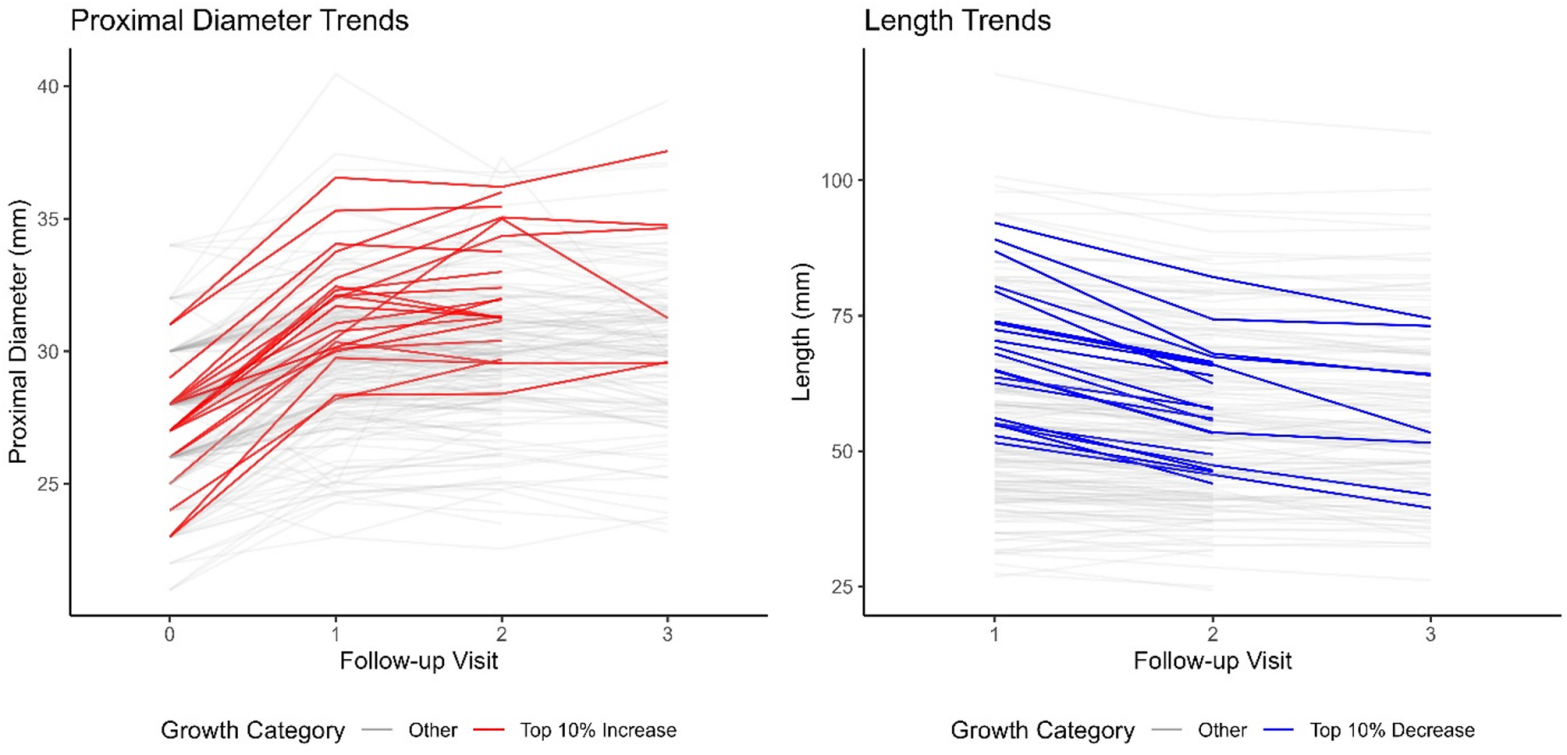
Spaghetti Plot visualizing measurements in mm against number of follow-up visit. Each line represents an individual patient. Highlighted lines depict the top 10% proximal diameter increases (red) or length decrease (blue)

In this cohort, our median follow-up was 33.38 months (21.36– 49.2). During this time, proximal median Dacron graft diameter increased by 2.03 mm (1.26–3.85). While not significant the linear mixed effects model, there was an absolute increase in distal diameter by 2.25 mm (1.26–3.99), when comparing median initial diameter, with diameter at the last follow-up. During this time, prosthesis length decreased by 3.20 mm (-6.94– -1.03). In total, 17 (7.9%) cases of anastomotic aneurysm and 21 (9.8%) cases of re-do surgery were noted. Table 3 depicts characteristics of the postoperative follow-up. Figure 1 illustrates absolute diameter over time during follow-up. A patient level spaghetti plot to illustrate individual trends can be found in the supplemental materials (Supplemental Fig. 1). Figure 2 depicts the 10% of patients with the highest increase in proximal diameter, as well as the highest decrease in length. Both boxplot and spaghetti plot show, that geometry changes mostly in during the first follow-up interval which includes the first 6–12 months after surgery.

**Table 3:** Postoperative outcomes; IQR: interquartile range

Characteristic	*N* = 215^*1*^
Difference in proximal diameter at FU 1 (mm)	2.00 (1.30, 3.38)
Difference in proximal diameter at FU 2 (mm)	2.20 (1.38, 3.75)
Difference in proximal diameter at FU 3 (mm)	2.03 (1.26, 3.85)
Difference in distal diameter at FU 1 (mm)	2.10 (1.25, 3.55)
Difference in distal diameter at FU 2 (mm)	2.40 (1.40, 3.80)
Difference in distal diameter at FU 3 (mm)	2.25 (1.26, 3.99)
Difference in length at FU 2 (mm)	-2.20 (-4.98, -0.35)
Difference in length at FU 3 (mm)	-3.20 (-6.94, -1.03)
Length of first follow-up (years)	0.02 (0.02, 0.29)
Length of second follow-up (years)	0.91 (0.50, 1.61)
Length of third follow-up (years)	2.78 (1.80, 4.02)
Anastomotic aneurysm	17 (7.9%)
Additional dissection	6 (2.8%)
Overall re-do surgery	21 (9.8%)
^*1*^Median (IQR); n (%)	

Figure

### Linear mixed effects model

**Fig. 3:**
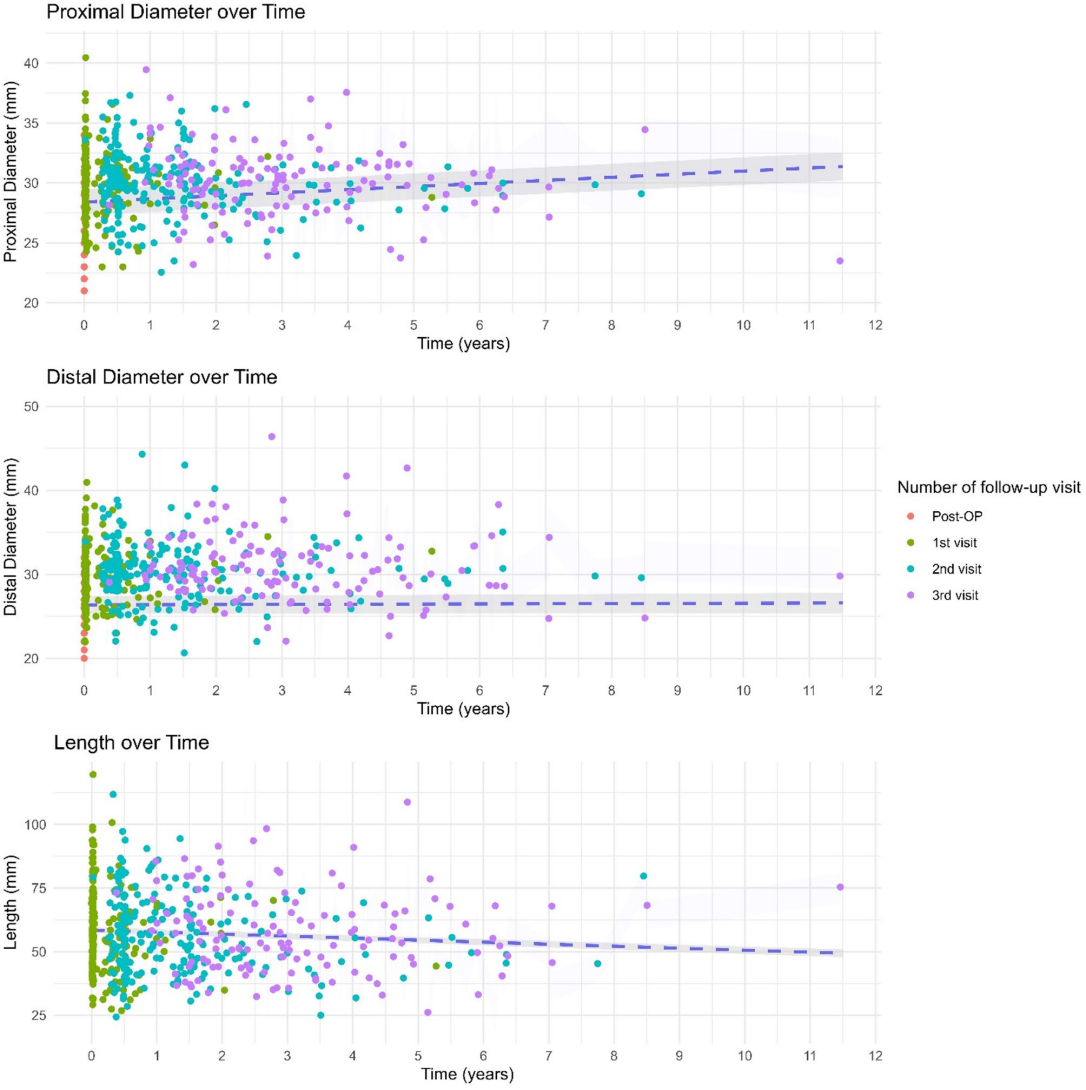
Scatter plots visualizing measurements over time in years after surgery. Colour differentiates between number of follow-up visit. A regression line was fitted with a 95% Confidence interval

Analysis of relationship aortic prostheses measurements over time yielded significant results regarding proximal diameter. Over a total follow-up of 11.5 years, proximal diameters increased significantly (*p* < 0.001). Estimated yearly growth was 0.251 cm / year (95%CI 0.181–0.328). No significant changes in distal diameter were noted during the reported follow-up period (*p* = 0.366). Estimates for yearly changes range from − 0.048 cm / year to 0.135 cm / year. Regarding Dacron graft length, we found significant yearly shortening (*p* < 0.001) of -0.835 cm/y. These results were visualized in a scatter plot with a linear regression line in Fig. 3.

## Discussion

This study’s key findings can be summarised as follows:

(I) Proximal, but not distal diameter of implanted ascending aortic Dacron grafts undergoes significant enlargement during postoperative follow-up.
(II) In contrast, prosthesis length decreases during this time.
(III) Preoperative planning of later endovascular aortic surgery should not rely solely on nominal diameters of implanted Dacron grafts but should be cognisant of the dynamic nature of Dacron prosthesis geometry.

### Comparison of patient cohort to literature

Baseline characteristics, operative characteristics and postoperative outcomes are generally comparable to aortic patients found in literature. Although the median age of our cohort was comparable to what is found in literature, some centres quote slightly higher numbers. This might be due to different scope of surgery offered at centres with a different level of expertise, shifting the age of the average patients [7–10]. As expected, a vast majority of our patient were previously diagnosed with hypertension, which is known to be a core predisposing factor for aortic dilation and dissection [2]. Consequently, high blood pressure might also be related to aortic graft diameter and length changes. Additionally, it is recommended to reduce postoperative hypertension to a minimum through meticulous medical therapy to avoid complications leading to re-do surgery. In our cohort, cases of re-do surgery and anastomotic aneurysm were both lower than commonly reported. This might be due to the high volume of open aortic cases at our centre, leading to generally more favourable outcomes [11].

(I) Our data shows, that over time, proximal diameter increases in Dacron grafts which were implanted in the ascending aorta, whereas distal diameter remains relatively unchanged. Similar data on the enlargement of aortic grafts has been previously published [3, 12]. However, a difference between proximal and distal Dacron graft growth has not been described before.

### Mechanistic interpretation

The mechanism responsible for this growth pattern could be associated with the loss of hydrostatic pressure found in straight tubes over their length. This may be caused by loss of energy due to viscous dissipation and friction from proximal to distal [13]. It is known that hypertension, and therefore elevated hydrostatic pressure in the vascular system, is one of the most prominent risk factors for the development of vascular aneurysms [2]. In turn, local pressure differences might be causative of dissimilar diameter growth depending on the location on the aortic graft. Additionally, the differences might be explained by the nature of the surrounding aorta. Proximal segments of the aorta are more elastic due to a different composition of the extracellular matrix [14, 15]. Therefore, radial stability around the anastomosis might differ between proximal and distal segments, which might be the cause for the differences in dilatation.

(II) Secondly, our data suggests that over time, the aortic prosthesis experiences a shortening. In current guidelines for the treatment of aortic disease, the length of the aorta, especially the ascending aorta, has gained recognition as a well-established predictive marker for adverse events, and was even included as an indication for surgery [2].

### Mechanistic interpretation

The shortening of the graft seen in our data might result from an overall increase in length of surrounding aortic segments. As woven and knitted Dacron grafts are generally more rigid than the aorta they are connected to, there might be an imbalance in deformation when comparing aortic tissue to graft material. Therefore, an even distribution of geometry changes such as lengthening is improbable, and the result is an apparent shortening of the aortic graft.

Previous studies have reported that native aortic tissue can exhibit post-surgical dimensional changes, including gradual dilation or elongation, depending on factors such as the underlying pathology and surgical technique. For example, the growth rate of the native aortic root has been shown to increase by approximately 0.1 mm/year in some cases [16]. However, Dacron grafts are composed of synthetic materials with distinct biomechanical properties, which likely account for the differences in behaviour observed in our study, such as proximal dilation and overall shortening of the graft. Future studies directly comparing graft and native aortic tissue changes could provide valuable insights into these dynamics and inform surgical planning and long-term follow-up strategies.

### Clinical implications

(III) Previously described results have important implications for the adequate planning of aortic repair during follow-up. Especially endovascular procedures profit immensely from accurate preoperative planning and understanding of the patient’s aorta and landing zones to ensure adequate oversizing and stability of the stentgraft [2, 17, 18]. As they offer superior radial stability, surgically implanted Dacron grafts are readily used as landing zones [2, 19]. With the lack of diameter increase in the distal segment of implanted Dacron grafts, our data supports their use as Zone 0 landing zones in thoracic endovascular repair including the aortic arch. However, changes in graft length need to be monitored tightly for future stentgraft treatment, as landing zones might shift and aggravate the risk of endoleaks. The observed dilation at the proximal Dacron graft segment should also be noted, as it might lead to an increased rate of type Ia endoleak if not considered when planning the oversizing of the stentgraft in the ascending aorta.

The potential mechanisms of this proximal dilatation raise an important consideration regarding graft oversizing during implantation. If the observed dilation is driven by radial strain exerted by the proximal native aortic segment, oversizing the graft may help mitigate these changes by accommodating the mechanical forces at play. However, if the dilation is primarily caused by hemodynamic strain acting directly on the graft material, independent of native aortic segment geometry, oversizing would not prevent these changes and could potentially introduce other complications. This highlights a critical challenge in determining optimal graft sizing, as understanding the underlying mechanisms of dilation—whether due to native aortic interactions or intrinsic hemodynamic forces—is essential for improving long-term outcomes and reducing graft-related complications.

Mitigating dimensional changes in Dacron grafts, such as proximal dilation and length shortening, is critical for improving long-term outcomes following aortic surgery. Rigorous postoperative blood pressure control through medical therapy has been shown to reduce hemodynamic stress on vascular structures and may help prevent proximal dilation and associated complications, such as anastomotic aneurysms [2, 3]. Additionally, surgical techniques that optimize graft sizing during implantation and ensure adequate radial stability are essential. For instance, methods such as axial stretching or flattening of graft crimps have been demonstrated to improve the dimensional stability of Dacron grafts under systemic pressures, potentially reducing early structural changes [20]. These considerations highlight the need for future prospective studies to evaluate the efficacy of these strategies in preventing graft-related complications and improving durability.

Based on our retrospective results, while we cannot give specific recommendations on oversizing- or other treatment strategies, we can summarize the following: Based on our data, when planning an endovascular procedure with a landing zone in a previously implanted polyester graft, all previous CTA scans should be considered, differences in diameter and length over time calculated, and sizing strategy adjusted accordingly. As seen in our data, significant changes of graft geometry should be expected in the first year of follow-up, as such larger oversizing might be advisable. In later interventions, more conservative oversizing may be feasible.

### Limitations and future directions

Our study is limited by multiple factors. Firstly, as our data was analysed retrospectively, results should be interpreted descriptively. Moreover, time intervals between follow-up visits are highly heterogeneous. Therefore, noise is introduced to our data. Additionally, for later timepoints there was a high rate of loss to follow-up, which renders making precise statements regarding later timepoints during follow-up difficult. Lastly, this analysis does not differentiate between dacron graft diameter and length changes regarding manufacturer of aortic prosthesis. As there is a possibility for heterogeneous geometry changes depending on the composition of the graft material, this should be investigated in future work.

Additionally, while the precision of measurements using multiplanar reconstruction (MPR) tools may be subject to limitations, our study employed standardized imaging protocols to minimize variability and ensure reproducibility. However, there is still a possibility for interobserver variability in the aortic measurements even with the use of these precise MPR tools. Due to the nature of our data collection, where each datapoint was documented be the same observer, analysis of this interobserver variability will not be feasible.

The observed proximal diameter growth rate of 0.25 cm/year reflects a statistically significant trend during follow-up but does not necessarily imply indefinite or exponential growth. Previous studies have demonstrated that initial expansion of Dacron grafts often diminishes over time due to stabilization of hemodynamic forces and material properties [2, 3, 21]. This highlights the importance of considering biological and mechanical factors, such as compliance mismatch and material fatigue, when interpreting long-term graft behaviour. Further research with extended follow-up is warranted to fully understand the trajectory of dimensional changes in Dacron grafts.

Measuring graft length presents unique challenges due to difficulties in defining clear boundaries, especially at the distal anastomosis where bias cuts are frequently employed during surgery. In our study, graft length was assessed by a single observer to reduce variability and ensure consistency. Despite these efforts, some degree of measurement uncertainty is unavoidable when evaluating graft geometry using imaging tools. Future studies may benefit from incorporating multiple observers or advanced imaging techniques to further refine measurement accuracy.

Lastly, the descriptive nature of this study precludes its results from being definitive. For the generation of robust recommendations, it is essential that future research investigates the clinical effects of different oversizing strategies when using polyester grafts as landing zones in a prospective manner.

## Conclusion

Our findings underscore the importance of accounting for dimensional changes in Dacron grafts, not only over time but also immediately after implantation. The difference between the nominal size of the graft and its pressurized size in vivo can significantly impact surgical planning, particularly in hybrid procedures and valve-sparing techniques. Surgeons should consider these changes when selecting graft sizes to ensure appropriate fit and long-term stability. This is especially critical when using Dacron grafts as landing zones for endovascular procedures or in complex repairs involving valve preservation. Future studies should further investigate this phenomenon to provide more detailed guidance on optimal sizing strategies.

## Electronic supplementary material

Below is the link to the electronic supplementary material.

Supplementary Material 1

## Data Availability

Due to restriction put forth by our local ethics committee, data may not be shared.

## Abbreviations

BMI: Body mass index
CTA: Computed tomography angiography
