# Supplementary material for: Morphological changes in polyester prosthesis geometry after open aortic repair
Source: BMC Cardiovasc Disord. 2025 Jun 2;25:426. doi: 10.1186/s12872-025-04851-0 (PMC12128550; doi:10.1186/s12872-025-04851-0)
Supplement: Supplementary file 1 — Supplementary Material 1 [file 12872_2025_4851_MOESM1_ESM.docx]

# Supplemental Methods

## Model robustness

| **Model** | **Outlier Analysis** | **Leverage Analysis** | **Robust** |
| --- | --- | --- | --- |
| Proximal diameter | 0.042cm – 0.080 cm | 0.053 cm – 0.10 cm | Yes |
| Distal diameter | -0.043 cm – 0.003 cm | -0.012 cm – 0.054 cm | Yes |
| Length | -0.19 cm - -0.097 cm | -0.099 cm - -0.054 cm | Yes |

Table S1. Analysis of model robustness. The outlier and leverage analyses are outlined in the methods section of the main body of the manuscript. The “Robust” column denotes, if the direction of the significant effect is the same in both robustness checks as in the main model.


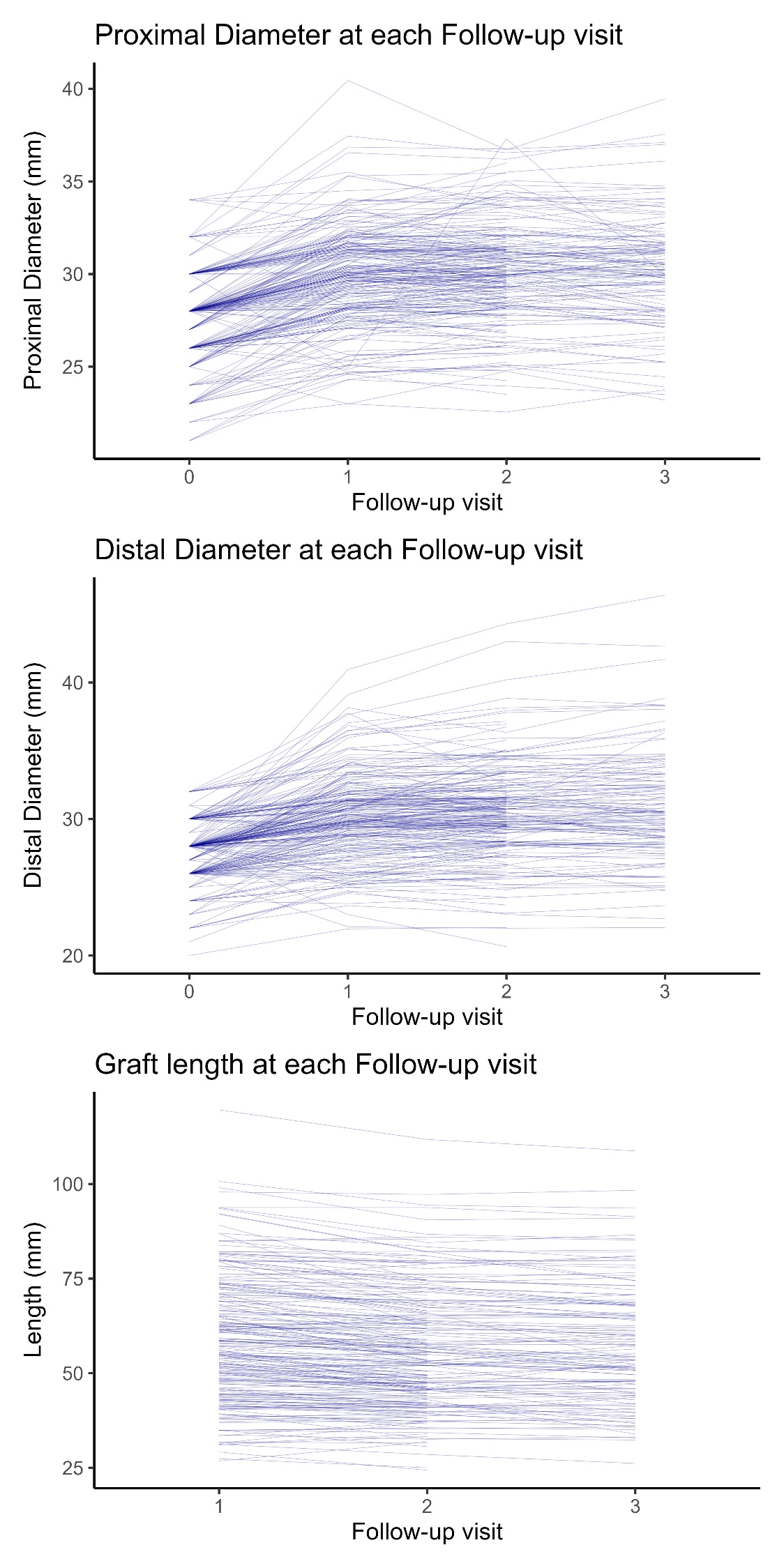


Supplemental Figure 1: Individual patient level spaghetti plot depicting diameters (mm) and length (mm) over time.
